# Adaptive variation for growth and resistance to a novel pathogen along climatic gradients in a foundation tree

**DOI:** 10.1111/eva.12796

**Published:** 2019-04-15

**Authors:** Collin W. Ahrens, Richard A. Mazanec, Trudy Paap, Katinka X. Ruthrof, Anthea Challis, Giles Hardy, Margaret Byrne, David T. Tissue, Paul D. Rymer

**Affiliations:** ^1^ Hawkesbury Institute for the Environment Western Sydney University Penrith New South Wales Australia; ^2^ Biodiversity and Conservation Science, Bentley Delivery Centre Western Australian Department of Biodiversity, Conservation and Attractions Perth Western Australia Australia; ^3^ Centre for Phytophthora Science and Management, School of Veterinary and Life Sciences Murdoch University Murdoch Western Australia Australia; ^4^ Kings Park Science Department of Biodiversity, Conservation and Attractions Perth Western Australia Australia; ^5^ Present address: Department of Microbiology and Plant Pathology, Forestry and Agricultural Biotechnology Institute (FABI) University of Pretoria Pretoria South Africa

**Keywords:** adaptive capacity, *Eucalyptus* sensu lato, heritability, *Quambalaria* shoot blight, standing genetic variation, trait evolution

## Abstract

Natural ecosystems are under pressure from increasing abiotic and biotic stressors, including climate change and novel pathogens, which are putting species at risk of local extinction, and altering community structure, composition and function. Here, we aim to assess adaptive variation in growth and fungal disease resistance within a foundation tree, *Corymbia calophylla* to determine local adaptation, trait heritability and genetic constraints in adapting to future environments. Two experimental planting sites were established in regions of contrasting rainfall with seed families from 18 populations capturing a wide range of climate origins (~4,000 individuals at each site). Every individual was measured in 2015 and 2016 for growth (height, basal diameter) and disease resistance to a recently introduced leaf blight pathogen (*Quambalaria pitereka*). Narrow‐sense heritability was estimated along with trait covariation. Trait variation was regressed against climate‐of‐origin, and multivariate models were used to develop predictive maps of growth and disease resistance. Growth and blight resistance traits differed significantly among populations, and these differences were consistent between experimental sites and sampling years. Growth and blight resistance were heritable, and comparisons between trait differentiation (*Q*_ST_) and genetic differentiation (*F*_ST_) revealed that population differences in height and blight resistance traits are due to divergent natural selection. Traits were significantly correlated with climate‐of‐origin, with cool and wet populations showing the highest levels of growth and blight resistance. These results provide evidence that plants have adaptive growth strategies and pathogen defence strategies. Indeed, the presence of standing genetic variation and trait heritability of growth and blight resistance provide capacity to respond to novel, external pressures. The integration of genetic variation into adaptive management strategies, such as assisted gene migration and seed sourcing, may be used to provide greater resilience for natural ecosystems to both biotic and abiotic stressors.

## 1 INTRODUCTION

Healthy forests are resilient to biotic and abiotic stressors and are able to mitigate potential impacts from climate change through high diversity and the maintenance of complex ecosystem processes (Chapin et al., 2000). These forests include a mosaic of plant and animal assemblages, and successional patches representing all stages of the natural range of disturbance and recovery (Trumbore, Brando, & Hartmann, 2015). Yet, globally, forests are under pressure from introduced pests, diseases and human disturbances including climate change. Global trade and modification of natural ecosystems for primary production, and industrial and urban development, have created pathways that facilitate the establishment and spread of pests and diseases (Holdenrieder, Pautasso, Weisberg, & Lonsdale, 2004) and increased forest susceptibility to pathogens (Perkins & Matlack, 2002). Changes in climate have increased the susceptibility to forest collapse (Allen, Breshears, & McDowell, 2015; Anderegg et al., 2015), through exceeding physiological safety margins (Choat et al., 2012; Drake et al., 2015). Therefore, it is critically important to determine the adaptive capacity of plants to a varying combination of stressors to inform management strategies in maintaining forest diversity, function and resilience.

Under altered environmental conditions, plant species must acclimate, adapt, move or succumb to external pressures (Corlett & Westcott, 2013). Plants can respond to changes in their environment using strategies that are either ecological or evolutionary in nature (Anderson, Willis, & Mitchell‐Olds, 2011). Acclimation represents an organism's short‐term (within the lifetime of the organism) capacity to respond to different environments (Palacio‐López, Beckage, Scheiner, & Molofsky, 2015), but a species’ long‐term (among generations of the species) adaptive capacity depends on the species’ genetic variation (standing or acquired through migration or mutation) that increases its evolutionary potential and physiological tolerance to environmental stressors (Frankham, 2005; Reed & Frankham, 2003). From an evolutionary perspective, the phenotypic differences between populations along environmental gradients may result from directional selection imposed by contrasting environments, neutral evolutionary processes or both (Savolainen et al., 2011; Vitasse et al., 2014). Plant growth is considered to be an adaptive trait, and different growth strategies can be detected in contrasting environmental conditions (Arendt, 1997; Moles, 2018). Likewise, pathogen resistance has been shown to develop differentially in regions with contrasting environments (Burdon & Thrall, 2009). Trait variation across environmental gradients may, however, have inconsistent distributions due to a combination of genotypic, environmental and interactive components.

One method to tease apart the genetic and environmental components of trait variation is to use common garden experiments with multiple genotypes established in different environments, which can elucidate the proportional effects of genetic adaptation and phenotypic plasticity within a species (Lepais & Bacles, 2014; Vitasse et al., 2014). Common garden experiments minimize and statistically account for environmental variance enabling the estimation of genetically determined variation in complex traits (de Villemereuil, Gaggiotti, Mouterde, & Till‐Bottraud, 2015; Whitham et al., 2006). One measure, *Q*_ST_, estimates quantitative trait differentiation between populations, and comparison between *Q*_ST_ and *F*_ST_ (genetic differentiation between populations) can disentangle evolutionary forces (Whitlock, 2008). Trait variation associated with environmental gradients can provide further evidence of selection in the evolution of functional traits (Vilà‐Cabrera, Martínez‐Vilalta, & Retana, 2015). In addition, the proportion of total phenotypic variance in a population that is attributable to additive genetic variation (i.e., narrow‐sense heritability ${\hat{h}}^{2}$; breeding values) can be estimated from common garden experiments with known pedigrees.

The forests of southwest Western Australia (WA) are under pressure from pathogens and climate change (Fitzpatrick, Gove, Sanders, & Dunn, 2008; Matusick, Ruthrof, Brouwers, Dell, & Hardy, 2013; Shearer, Crane, Barrett, & Cochrane, 2007). The foundation tree, *Corymbia calophylla* (Lindl.) K. D. Hill & L. A. S. Johnson (*Eucalyptus* sensu lato; family Myrtaceae), is common throughout the Southwest Australia Biodiversity Hotspot. *Corymbia calophylla* is an economically and ecologically important tree for the forestry industry and biodiversity management. It is an ideal species to study adaptation and plasticity because (a) it spans several environmental transitions and has recently undergone climate‐induced episodic mortality events (Matusick et al., 2013); (b) genomic patterns of adaptation to climate have been identified (Ahrens et al., [Link]); and (c) there has been a relatively recent introduction of the basidiomycetes leaf blight (*Quambalaria pitereka* [Walker & Bertus] Simpson) to Western Australia (first described in WA in 1993) from eastern Australia where it is a major pathogen of species and hybrids in the *Corymbia* complex (Pegg et al., 2008). *Quambalaria pitereka* is of growing concern in WA because of the increased ubiquity and negative impact on forest stands (Paap, Burgess, McComb, Shearer, & Hardy, 2008). *Quambalaria* is a primary pathogen and affects new flush causing spotting, necrosis and distortion of expanding leaves and green stems, but very little is known of the biology of *Quambalaria* species (Pegg, Carnegie, Wingfield, & Drenth, 2009). *Quambalaria pitereka* reproduces quickly, creating sporulating lesions in 10–14 days under favourable conditions (Pegg, Webb, Carnegie, Wingfield, & Drenth, 2009), and is known to lead to loss in leaf area and change in tree canopy morphology (Pegg et al., 2008). Dispersal of *Q. pitereka* generally occurs through splash‐dispersal and wind‐driven rain (Pegg, Nahrung, Carnegie, Wingfield, & Drenth, 2011), but due to fast life cycles and tree density, >60% of a *Corymbia citriodora* subsp. *variegata* plantation was infected after only 87 days from first occurrence (Pegg et al., 2011). *Quambalaria pitereka* is widespread throughout the south‐west Australian forests and woodlands, although the distribution and abundance is currently unknown and is in the process of being defined through molecular markers and field surveys (Prof G. Hardy, personal communication).

It is predicted that Mediterranean climates, such as southwest Western Australia, will experience significant geographic contraction over the next few decades, driven by hotter and drier conditions (IPCC, 2013; Matesanz & Valladares, 2014), and increasing exposure to pest and disease (Holdenrieder et al., 2004; Juroszek & von Tiedemann, 2011). Our main objective is to investigate the adaptability of complex traits and provide insights into the ecological and evolutionary response to current and future environmental change. We used two experimental planting sites to elucidate the capacity of a foundational tree species to adapt to environmental pressures (disease and climate). We hypothesized (a) that growth traits were heritable and under selection pressure from their local environments. In contrast, because leaf blight and *C. calophylla* do not share a co‐evolutionary history, we hypothesized (b) that all populations to be equally susceptible to the pathogen and show limited heritability for pathogen resistance. As such, we aim to simultaneously clarify the heritability and covariation of growth and disease resistance traits of *C. calophylla* in order to incorporate this ecological and evolutionary knowledge into adaptive management strategies.

## 2 METHODS

### 2.1 Seed collection

**Figure 1:**
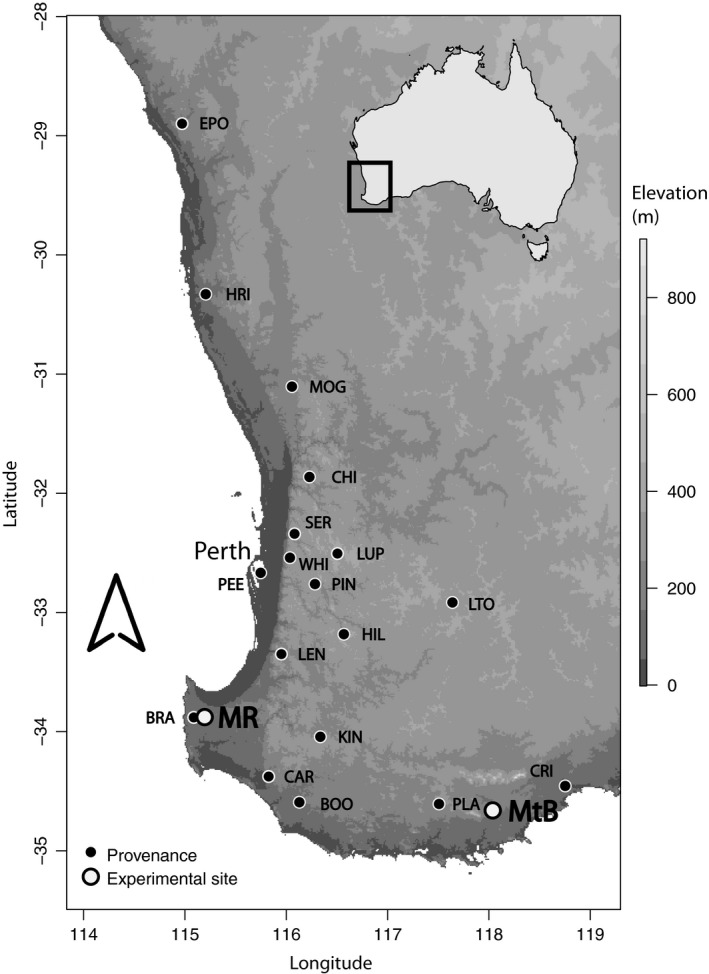
Map of southwestern Western Australia with population and experimental site locations and elevation (m)

Open‐pollinated seed families were collected from 18 naturally occurring populations of *C. calophylla* across the climatic and geographic distribution. Seed was collected by the Western Australian Department of Biodiversity, Conservation and Attractions (formerly the Department of Conservation and Land Management) between 1991 and 1992, and supplemented with seven additional populations including outlying populations in 2013 (Table 1 and Figure 1). Within each population, seed was sampled from c.10 parent trees that represent half‐sibling seed families in this outcrossing system. Trees were separated by >100 m to minimize relatedness due to neighbourhood effects. Fruit from individual seed‐lots was dried, and the extracted seed was stored in a cool room (temperature 1–3°C) to maintain high seed viability.

**Table 1:** Location and environmental information for the 18 populations used in the experimental sites

Population	Prov	MtB *F*	MR *F*	Latitude	Longitude	*T*_MAX_	MAP	1/AI
Ellendale pool	EPO	4	2	−28.859°	114.969°	34.1	467	3.33
Hill river	HRI	10	9	−30.311°	115.202°	31.7	563	2.56
Mogumber	MOG	10	10	−31.099°	116.051°	33.3	579	2.56
Chidlow	CHI	10	10	−31.868°	116.223°	32.2	900	1.54
Serpentine	SER	10	10	−32.353°	116.076°	30.5	1,173	1.12
Lupton	LUP	10	10	−32.521°	116.499°	31.6	635	2.22
Whittaker	WHI	10	10	−32.556°	116.031°	30.0	1,187	1.10
Peel Inlet	PEE	8	8	−32.685°	115.743°	30.4	885	1.49
Pindalup	PIN	10	10	−32.781°	116.278°	30.3	935	1.43
Lake Toolibin	LTO	10	10	−32.937°	117.632°	31.2	358	3.85
Hillman	HIL	9	9	−33.208°	116.562°	30.2	678	2.00
Lennard	LEN	10	10	−33.377°	115.947°	30.7	912	1.47
Bramley	BRA	9	9	−33.916°	115.083°	26.1	1,072	1.04
Kingston	KIN	10	10	−34.081°	116.330°	27.7	820	1.49
Carey	CAR	10	10	−34.420°	115.821°	25.9	1,106	1.02
Cape Riche	CRI	10	8	−34.602°	118.743°	26.2	579	2.08
Boorara	BOO	10	10	−34.639°	116.124°	25.6	1,159	0.95
Plantagenet	PLA	10	10	−34.653°	117.499°	26.7	733	1.59
Total/Mean		170	165			29.7	818.9	1.56

Note

Abbreviation(s): 1/AI: 1/Aridity Index; MAP: mean annual precipitation; MR *F*: number of open‐pollinated families at Margaret River; MtB *F*: number of open‐pollinated families at Mount Barker; *T*_MAX_: maximum temperature of the warmest month.

In 2014, seeds were germinated and grown in nursery trays (all seed‐lots had >80% germination). Seedlings were individually labelled and the trials pre‐assembled in the nursery using TrayPak, an Excel macro that translates the designed planting layout into an ordered series of nursery trays for efficient deployment of the trial in the field. Depending on seedling availability, some families were not equally represented on both sites (see Table 1). In 2014, seedlings were planted at trial sites using Pottiputki^®^ planting tubes shortly following deep ripping to a depth of 35–50 cm.

### 2.2 Trial design

Two sites, Mount Barker (MtB) and Margaret River (MR), Western Australia, were established in August and October of 2014, respectively. The trial locations are characterized by contemporary climates with different precipitation, but similar temperature regimes (Table S1). The MtB site was established within land that is managed as a *Eucalyptus* plantation; the MR site was embedded (and fenced) within a cleared area currently used for pasture grazing and comprised mainly of grass and forb species.

Eighteen populations represented by 170 and 165 families were established at MtB and MR (total of 4,080 and 3,960 trees, respectively). Row‐column designs with six blocks were generated using cycdesign (VSNi). At MtB, there were 15 rows and 11 columns within a block, while at MR there were 17 rows and 10 columns within a block. Families were randomly allocated to four tree row‐plots, replicated with six blocks, providing a total of 24 seedlings per family at each site. Spacing within a row was 2 m, and spacing between rows was 4 m on both sites. A double buffer row of seedlings was planted using the same spacing around each trial to minimize edge effects. The total area of the experimental sites was 38,800 m^2^ at MtB and 36,300 m^2^ at MR.

### 2.3 Measurements

Soil characteristics were determined at each site by removing ~1 kg of soil, 5 cm below the surface, at 3–6 random locations across each site and stored in separate cloth bags. A standard soil chemistry analysis was conducted at CSBP (Bibra Lake, Western Australia, Australia) on each sample and averaged within each site to estimate total pools of potassium (K), phosphorus (P), sulphur (S), nitrates and organic carbon.

Trees at both sites were measured in December 2015 and September 2016 (14–16 months and 23–25 months after planting, respectively). All trees were measured for *height*, basal diameter (*diameter*) and blight *(blight resistance*; see scoring system below). Tree height was measured with an extendable 5‐m measurement pole to the nearest 5 cm with independent sighting to validate the measurement. Many individual trees, particularly at MtB, were multi‐stemmed with basal diameters of tertiary stems under 2 cm. Therefore, *diameter* for each tree was calculated by taking the square root of the sum of squared diameters of all stems that were at least 75% of the largest stem. The *blight resistance* score (1–5) was assessed by visually determining the percentage of tree tips with evidence of blight (0% = 5; 1%–25% = 4; 26%–50% = 3; 51%–75% = 2; 76%–100% = 1), following the technique of Brawner, Lee, Hardner, and Dieters (2011).

### 2.4 Statistical analysis

#### 2.4.1 Growth and defence

There was concern that *height* differences among populations may be directly attributed to pathogen load. To determine whether height was impacted by pathogen load, we compared populations within each *blight resistance* category (1–5) by employing a linear model (function *lm*). This allowed us to directly compare populations within the same *blight resistance* category (i.e., removing blight as an independent variable from the statistical analysis). There were two analyses performed: first, comparisons were made between populations within each *blight resistance* category, and significant differences were tested using a *post hoc* Tukey's test in *R* with an alpha value of 0.05. If differences among populations within *blight resistance* categories remain consistent across *blight resistance* categories, then the pathogen does not affect population height ranks. Second, slopes among the blight resistance categories versus climate‐of‐origin (maximum temperature of the warmest month) were compared using the ANOVA function in R (R Core Development Team, 2015). If slopes among blight resistance categories are not statistically different, then blight has not affected population height ranks. This is important because if *height* ranks are different because of blight, then our growth results are differentially affected by blight, indicating that we would have to reconsider our *height* results. Means and standard errors were calculated for all populations within each experimental site for *height*,* diameter* and *blight resistance* from the September 2016 measurement (25 months old) using the *summaryBy* function in the *doBy* package in R. Mixed‐effects models with the *lme* function and block as a random effect were used in R to test differences between populations using the *anova.lme* function. Figures were developed in R using base plotting commands and the package *ggplot2* (Wickham, 2009).

#### 2.4.2 Heritability

Heritability and best linear unbiased prediction (BLUP) for *height*,* diameter* and *blight resistance* from the September 2016 measurement (when trees were 25 and 23 months old) were estimated using ASReml version 4.1 (Gilmour, Gogel, Cullis, Welham, & Thompson, 2014). Initial analyses were conducted on individual traits to assess model fit; details of model fit assessment are given in the Supporting Information.

Prior to analysis, data for each site were standardized following White, Adams, and Neale (2007). The purpose of standardization was to homogenize within block variances across blocks within a site and across sites, thereby removing scale effects from family x environment interaction.

Residual plots were examined for normality, and transformation was not necessary.

For multi‐site analysis, data for individual traits were analysed using a cross‐classified model with details given in the Supporting Information. In order to determine if there is a genotype × environment interaction, type‐B cross‐site correlations (Burdon, 1977) were estimated for family effects according to the formula defined in White et al., (2007) and details are given in Supporting Information.

Within population heritability was estimated from variance components according to the formula:

$${\hat{h}}^{2}=\frac{\sigma_{\text{ad}}^{2}}{\sigma_{f}^{2}+\sigma_{b}^{2}+\sigma_{e}^{2}}$$

and for cross‐site heritability, the formula:

$${\hat{h}}^{2}=\frac{\sigma_{\text{ad}}^{2}}{\sigma_{f}^{2}+\sigma_{\text{sf}}^{2}+\sigma_{\text{sb}}^{2}+\sigma_{e}^{2}}$$

where ${\hat{h}}^{2}$ is the narrow‐sense heritability; $\sigma_{\text{ad}}^{2}$ is the additive genetic variance component estimated from the family variance component ($\sigma_{f}^{2}$) by multiplying it by 2.5 (i.e., $\sigma_{\text{ad}}^{2}=2.5\;\times \;\sigma_{f}^{2}$; details below); $\sigma_{b}^{2}$ is the block component of variance, where block represents family × block interaction; $\sigma_{e}^{2}$ is the error component of variance; $\sigma_{\text{sb}}^{2}$ is the block within site component of variance; and $\sigma_{\text{sf}}^{2}$ is the family × site interaction component of variance. Mixed‐mating systems in open‐pollinated eucalypts may produce inflated heritability estimates for growth traits (Costa e Silva, Hardner, & Potts, 2010; Griffin & Cotterill, 1988; Hodge, Volker, Potts, & Owen, 1996); therefore, a coefficient of relationship of *ρ* = 1/2.5 was applied when estimating the additive variance component to compensate for selfing rates of about 30% (Griffin & Cotterill, 1988). This coefficient was appropriate for first‐generation eucalypt progeny, suitably correcting variance components and heritability estimates (Bush, Kain, Matheson, & Kanowski, 2010).

Phenotypic and genetic correlations among traits were calculated according to the standard formula for correlation:

$$r=\frac{\sigma_{12}^{2}}{\sqrt{\sigma_{1}^{2}*\sigma_{2}^{2}}}$$

where *r* is the correlation coefficient, $\sigma_{12}^{2}$ is either the additive genetic covariance or the phenotypic covariance, and $\sigma_{1}^{2}$ and $\sigma_{2}^{2}$ are additive genetic variances or total phenotypic variances. Additive genetic variances and covariances were calculated as $\sigma_{f}^{2}$ *2.5 and $\sigma_{12}^{2}$ *2.5 respectively, where $\sigma_{12}^{2}$ = the covariance between two traits.

To test for divergent natural selection on each of the three traits, we compared Q_ST_ and F_ST_ values using the *QstFstComp* package in R (https://github.com/kjgilbert/QstFstComp), which is based on the methods outlined in Gilbert and Whitlock (2015). This package explicitly compares population differentiation of quantitative traits to neutral genetic differentiation. It employs parametric resampling of *Q*_ST_ and bootstraps across single nucleotide polymorphisms (SNPs) to estimate predicted neutral *Q*_ST_ and the uncertainty of Weir and Cockerham's (1984) *F*_ST_. Genotyping discovery and bioinformatics is described in Supporting Information. In brief, genetic differentiation was estimated from 9,560 SNPs among 18 populations with 10 individuals using a reduced representation sequencing approach similar to RADseq. The QstFstComp function provides estimated *Q*_ST_ and *F*_ST_ values along with statistical test of the difference between *Q*_ST_ − *F*_ST_ and neutral *Q*_ST_ − *F*_ST_. This test was performed for each trait from both experimental sites using the following conditions: 10,000 simulations and half.sib.dam model. The half.sib.dam model meets our criteria that all sampled half‐siblings are from dams nested within populations and sired by separate and unknown individuals.

#### 2.4.3 Trait–Environment correlations

Population trait means were plotted against environmental variables from their origin using R to understand if temperature or precipitation explained the more variation in a univariate framework. Average maximum temperature of the warmest month (*T*_MAX_) and precipitation of the driest month (*P*_DM_) were extrapolated from WorldClim data sets (www.worldclim.org) for each population (Table 1) using QGIS V2.14 (Quantum GIS Development team). *T*_MAX_ and *P*_DM_ were chosen because they represent the extreme temperature and precipitation variables and they had greater explanatory power compared to all other temperature (e.g., ∆AIC for *height* = 27.6 for *T*_MAX_ vs. 32.0–55.9 for other temperature variables) and precipitation (e.g., ∆AIC *height* = 29.0 for *P*_DM_ vs. 31.8–53.2 for other precipitation variables) variables from Worldclim. The aridity index (AI) raster was downloaded from CGIAR‐CSI (http://www.cgiar-csi.org/) and was transformed to 1/AI throughout the manuscript for greater clarity (i.e., more arid regions have a larger number). Linear models were used to define the line of best fit among population means, calculate the *r* ^2^ value and extract a *p*‐value by employing the *lm* function in R. Slopes between experimental sites were tested in the *lm* model by recording the interaction term between site and climate‐of‐origin with the trait as the response variable.

We also used multivariate modelling methods to understand how a combination of climate variables could explain the patterns we uncovered. Data from September 2016, representing 23–25 months cumulative growth and pathogen load, were normalized within each trial ((*x* − min)/(max − min)). Normalization of data from both experimental sites made it comparable and allowed it to be combined for model calculations. We used a multivariate generalized linear model to understand the relationship between environments and traits. Each of the three traits (*diameter*,* height* and *blight resistance*) was modelled against all possible combinations of 14 different climatic variables extracted from worldclim and CGIAR‐CSI Global‐Aridity and Global‐PET Database (http://www.cgiar-csi.org/). Twelve bioclim variables (Annual Mean Temperature; Temperature Seasonality; Max Temperature of Warmest Month; Min Temperature of Coldest Month; Mean Temperature of Wettest Quarter; Mean Temperature of Driest Quarter; Annual Precipitation; Precipitation of Wettest Month; Precipitation of Driest Month; Precipitation Seasonality; Precipitation of Warmest Quarter; and Precipitation of Coldest Quarter) were extracted using the getData and extract functions in the R package “*raster*” from 2.5 resolution maps. Likewise, aridity index and potential evapotranspiration (PET) were downloaded directly from CGIAR‐CSI. All combinations were extracted using the *dredge* function in the *MuMIn* package. Each model was compared and ranked according to AICc. The three optimal models with the lowest AICc are reported. The variance explained for all three optimal models was estimated using the *Dsquared* function in the *modEvA* package.

In addition to the above complex model, a simpler model was developed based on a few key climatic variables in an attempt to provide a more interpretable output. The three key explanatory variables (*T*_MAX_, *P*_DM_ and AI) were modelled against the three dependent variables (*height*,* diameter* and *blight resistance*) and ranked using AICc in the same manner described above. The variance explained was calculated using the *Dsquared* function in the *modEvA* package for each GLM model. The intercept and beta coefficients (partial regression coefficients) were recorded and used along with the three environmental raster layers in the QGIS raster calculator to visualize the distribution across the landscape of the three traits.

## 3 RESULTS

### 3.1 Site characteristics

Soil at each experimental site varied markedly in the availability of nitrates, phosphorus (P), potassium (K), sulphur (S) and organic carbon (Table S2). After 25 months, successful establishment varied between the experimental sites; 3,572 of 4,080 trees in MtB (87.5%) and 3,714 of 3,960 trees in MR (93.8%) survived. Mortality at the MR site was largely directly attributable to *Phytophthora* (*P. ornamentata* and *P. crassamura*; collections were cultured for identification) and African Black Beetle (*Heteronychus arator*). *Phytophthora* damage was limited to a portion of the experimental site, which showed evidence of poor water drainage. Mortality at the MtB site was sporadic and mostly attributable to a high weed load, particularly red ink plant (*Phytolacca octandra* L.), which outcompeted some of the target experimental trees.

### 3.2 Traits

Best linear unbiased predictions (BLUPs Table S2) for population *height* at ages 23–25 months ranged from 205.4 cm ± 3.9 (*SE*) to 280.3 cm ± 3.9 at MtB and from 100.8 ± 4.0 to 155.1 ± 3.9 at MR. Population estimated BLUPs for basal *diameter* ranged from 5.63 ± 0.14 cm to 7.51 cm ± 0.11 at MtB and from 2.86 cm ± 0.15 to 3.88 cm ± 0.11 at MR. Population estimated BLUPs for *blight resistance* scores ranged from 2.0 ± 0.1 to 3.3 ± 0.1 at MtB and 2.6 ± 0.1 to 4.0 ± 0.1 for MR. Leaf blight incidence was similar between sites with 93.6% (3,416 of 3,651) of the plants infected at MR and 97.8% (3,481 of 3,561) of the plants infected at MtB. Neither statistical test employed showed blight affecting height ranks (except for blight resistance category 1), as slopes between blight categories 2 through 5 and the overall data were not statistically different (*p* > 0.05) and population differences within categories remained consistent, with the one population exception being LTO, which had high standard errors due to low numbers (Table S3 and Figure S1). The slopes from the most severely blight afflicted trees (*blight resistance* index of 1) were significantly different than all other slopes (*p* < 0.05), and the slopes were not statistically different than 0 (*p* > 0.05; Table S3 and Figure S1). This indicates that, other than the most severely afflicted trees, differences among populations for the *height* trait were real and not artificially inflated by blight.

Populations exhibited significant differences in growth and *blight resistance* (Figures S2 and S3), and these differences were consistent among experimental sites. There were significant differences in *height* between populations both within and across sites (*p* < 0.001; Figure S3). *Diameter* was also significantly different at the population level across experimental sites (*p* < 0.001) and within each experimental site (*p* < 0.001 for both sites). *Blight resistance* was significant at the population level across experimental sites (*p* < 0.001) and within experimental sites (*p* < 0.001 for both sites).

### 3.3 Genetic parameters

Narrow‐sense heritability estimates for *height* and *diameter* were similar between the two sites (Table 2). *Blight resistance* heritability was different among sites (Table 2). Cross‐site heritability was similar for all traits (Table 2). Phenotypic correlation (*r*_p_) was high between the growth traits, but low between *blight resistance* and growth traits (Table 2). The genetic correlation (*r*_g_) follows the same pattern except for the blight/height correlation, which was 0.46 in MtB and 0.54 in MR. Type‐B genetic correlations for all three traits were >0.90, indicating the absence of significant G x E interaction across experimental sites.

**Table 2:** Population narrow‐sense heritability (grey diagonal) with phenotypic (*r*_p_; below diagonal) and genetic (*r*_g_; above diagonal) correlations from the 2016 measurements. Standard error is given. (a) Margaret River and (b) Mount Barker

(a)	Height	Dia	Blight	(b)	Height	Dia	Blight
Height	0.14 ± 0.03	0.70 ± 0.09	0.58 ± 0.21	Height	0.18 ± 0.04	0.8 ± 0.07	0.45 ± 0.13
Dia	0.77 ± 0.01	0.12 ± 0.03	0.14 ± 0.23	Dia	0.74 ± 0.01	0.11 ± 0.03	0.13 ± 0.17
Blight	0.00 ± 0.02	‐0.14 ± 0.02	0.08 ± 0.03	Blight	0.22 ± 0.02	0.09 ± 0.02	0.19 ± 0.04

Note

Abbreviation(s): Blight: blight resistance; Dia: basal diameter.

Quantitative trait differentiation (*Q*_ST_; Table 3) minus genetic differentiation (*F*_ST_: Table S4) was significantly greater than the neutral resampling of *Q*_ST_ − *F*_ST_ for *height* and *blight resistance* at both experimental sites, but not different for *diameter* at either site (Table 3). Trait differentiation between populations was greatest for *height* (*Q*_ST_ = 0.27 [MR] and 0.12 [MB]) and *blight resistance* (*Q*_ST_ = 0.18 [MR] and 0.19 [MB]), and lowest for *diameter* (*Q*_ST_ = 0.08 [MR] and 0.03 [MB]).

**Table 3:** Population‐level quantitative trait differentiation (QST)

	Trait	*Q*_ST_ (95% CI)	Neutral *Q*_ST_ (resampled)	Upper one‐tailed *p*‐value	*Q*_ST_ − *F*_ST_
Margaret River	Height	**0.27** (0.13–0.43)	0.06	<0.001	0.21
	Diameter	0.08 (0.02–0.17)	0.06	0.39	0.03
	Blight	**0.18** (0.08–0.33)	0.06	<0.001	0.12
Mount Barker	Height	**0.12** (0.04–0.23)	0.06	0.03	0.06
	Diameter	0.03 (−0.001–0.08)	0.06	0.84	−0.03
	Blight	**0.19** (0.08–0.33)	0.06	0.001	0.13

Note

Global *F*_ST_ among all 18 populations was 0.057. Upper one‐tailed *p*‐values describe significantly greater differences of measured *Q*_ST_ − *F*_ST_ than 0 (denoted by bold *Q*_ST_ values), providing evidence for population‐level divergent selection.

### 3.4 Trait–environment correlations

**Figure 2:**
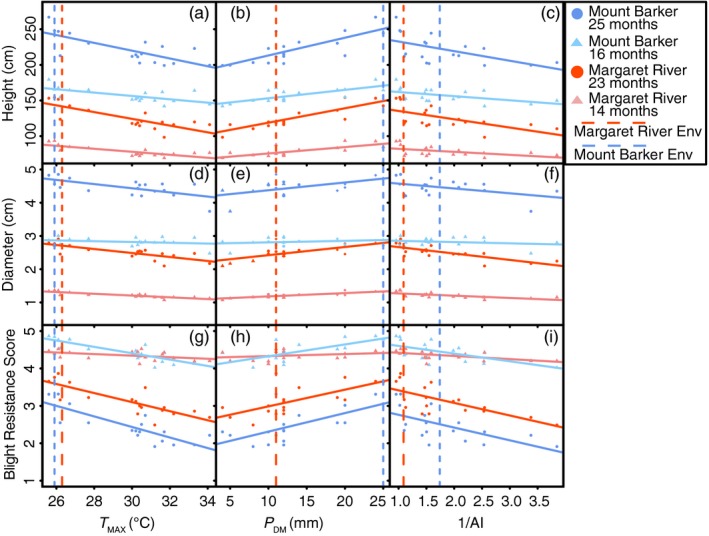
Population trait means plotted against environment‐of‐origin. Regressions between trait characteristics of *height* (cm^3^; a–c), *diameter* (d–f) and *blight resistance* (g–i) and three environmental variables (maximum temperature of the warmest month [*T*_MAX_; a, d, g], precipitation of the driest month [*P*_DM_; b, e, h] and 1/Aridity Index [AI; c, f, i]). The environment recorded (Env) for both experimental sites is given by the vertical dotted lines. *Height*,* diameter* and *blight resistance* are shown with two separate ages. All regression lines are significant (*p* < 0.05), showing that all three environmental factors significantly explain population variation in the three traits. The *r* ^2^ and *p*‐values are given in Table S5

All relationships between three environmental variables (*P*_DM_ *, T*_MAX_ and AI) and three traits (*height*,* diameter, and blight resistance*) across sites and years showed significant linear relationships (Figure 2), except for four correlations in 2015 between *diameter* and *P*_DM_, *diameter* and *T*_MAX_, and *diameter* and AI at MtB, and *blight resistance* and *P*_DM_ at MR (Table S5). The trait–environment relationships with the greatest *r* ^2^ value were consistently associated with *T*_MAX_ (Figure 2 and Table S5). The relationship between experimental sites at 23–25 months was not significantly different for any of the three climate‐of‐origin variables (*T*_MAX_: *blight resistance p* = 0.56, *height p* = 0.64 and *diameter p* = 0.91; *P*_DM_: *blight resistance p* = 0.80, *height p* = 0.51 and *diameter p* = 0.92; AI: *blight resistance p* = 0.74, *height p* = 0.74 and *diameter p* = 0.42), indicating that the trait responses (slope) among sites were not different. Therefore, the population by experimental site (GxE) interaction was not significant. In addition, the slopes of traits between years were not significantly different (*p* > 0.05) for all traits and experimental sites, except for *blight resistance* in MR.

The optimal complex models for *height* and *diameter* indicated that a combination of precipitation‐related variables explained the most variation across the landscape (Table S6), whereas the optimal model for *blight resistance* included four precipitation and three temperature variables. AI was only significant for the complex *height* model. The optimal models explained 86.0% of the variation for *height*, 72.7% for *diameter* and 91.6% for *blight resistance* (Table S6).

**Figure 3:**
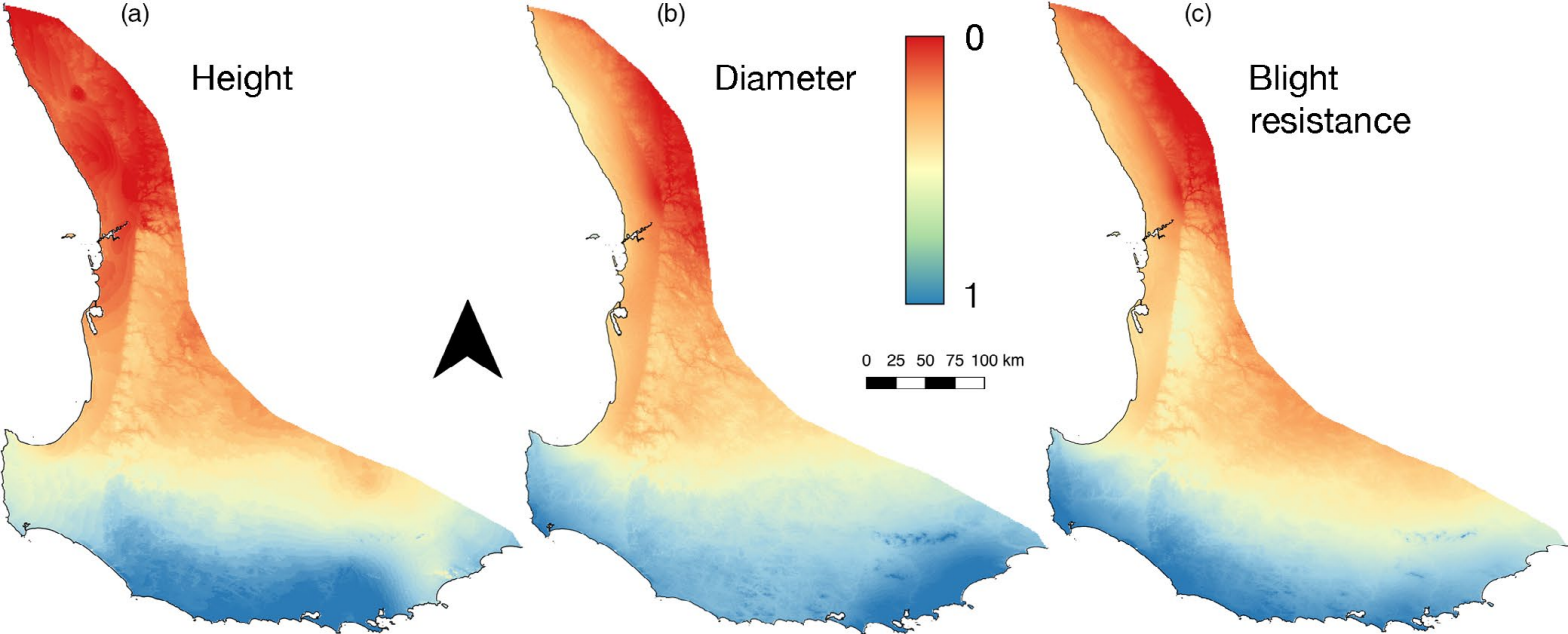
Predicted distribution of relative (a) *height*, (b) *diameter* and (c) *blight resistance* across the natural range of *Corymbia calophylla*. Normalized data were used to incorporate data from both experimental sites in 2016, red is interpreted as smaller (a & b) or less resistant to blight (c), and blue is interpreted as larger (a & b) and more resistant to blight (c). Models were created using the beta coefficients for maximum temperature of the warmest month with aridity index (a) or precipitation of the driest month (b & c) from Table 4. Refer to Figure 1 for orientation in Australia

The less complex models with three climatic variables explained between 52.9% and 72.1% of the trait variation (Table 4). For all of three traits, only two independent variables were significant. The less complex models were used to visualize the distribution of traits across the landscape (Figure 3). The distribution for all three traits showed similar change between the southern (faster growth and more pathogen resistance) and the northern portion of the distribution (slower growth and less pathogen resistance).

**Table 4:** Generalized linear models with standardized data from both experimental sites

	Intercept	*T*_MAX_	*P*_DM_	1/AI	VE
Height	1.619	−0.05	0.02	ns	57.7%
Dia	2.812	−0.05	ns	−0.09	52.9%
Blight	2.895	−0.08	ns	−0.09	72.1%

Note

Beta coefficients for each environmental variable and VE.

Abbreviation(s): 1/AI: 1/Aridity Index; Blight: *blight resistance*; Dia: basal diameter; ns: not significant; *P*_DM_: Precipitation of Driest Month; *T*_MAX_: Max Temperature of Warmest Month; VE: variation explained.

## 4 DISCUSSION

Our evaluation of the environmental and genetic determinants of growth and pathogen resistance in the foundation tree, *Corymbia calophylla,* found that these complex traits are heritable, indicating that this species has the genetic architecture required for trait evolution driven by natural (or human directed) selection. In addition, populations from wetter, cooler climates (located in the south) have adopted a faster growth strategy with more effective *blight resistance* than the populations from warmer and/or drier regions (located in the north) which have a slower growth strategy. We discuss the variance components of these complex traits, environmental associations and possible management strategies to incorporate adaptive capacity into the system.

### 4.1 Traits

Populations exhibited significantly different growth rates and *blight resistance* under two contrasting precipitation conditions measured across 2 years. While the trait values across years were different (as expected), the trends associated with climate‐of‐origin were consistent, providing confidence in the results. The trends among populations remained constant across experimental sites (i.e., same slope, different intercept) in accordance with type‐B genetic correlations, indicating there was no genotype by environment interactions. Populations from warmer climates grew slower and exhibited a lower resistance to blight in both experimental planting sites compared to faster growing, more resistant populations from cooler climates.

Correlations between growth and *blight resistance* are similar to other studies exploring the effect of *Q. pitereka* on forest plantations in the pathogen's native range in eastern Australia (Brawner et al., 2011; Johnson, Carnegie, & Henson, 2009). However, we point out that the correlation at the population level is not necessarily representative of a causal relationship. While Brawner et al. (2011) found that blight impacts height for *C. citriodora*, we found that blight only impacts height ranks in *C. calophylla* when the blight pathogen damage is most severe. Our results suggest that the *height* differences among populations are present if blight damage remains below 75% tree coverage. In other words, patterns of growth associated with climate‐of‐origin are not due to leaf blight. In fact, these results suggest that leaf blight would equalize growth rates across populations, not differentiate them.

The two traits, *height* and *blight resistance*, have very similar distributions with positive *r*_g_ and *r*_p_. Yet, these traits do not appear to be genetically linked. There are strong correlations between *height* and *blight resistance* at the population and family level, but this relationship is not present within each *blight resistance* category, except for the most affected trees. At the MR site, the slightly negative or neutral *r*_p_ combined with a positive *r*_g_ between *diameter* (negative *r*_p_) and *blight resistance*, and height (neutral *r*_p_) and *blight resistance* indicates that strong environmental sources of variation affect the traits (Falconer & Mckay, 1996). However, both *r*_p_ and *r*_g_ for *height*,* diameter* and *blight resistance* at MtB are positive, indicating that genetic variation strongly contributes to phenotype. Because of this complex relationship, we are unable to rule out pleiotropy and/or linkage disequilibrium effects between the two traits and further experiments would have to be performed to identify causal genes and quantify their possible overlapping functions.

Growth characteristics in *C. calophylla* follow previous results showing increased growth among cool climate‐of‐origin populations in field sites (O'Brien & Krauss, 2010) and glasshouse experiments (Rymer, P.D., Ahrens, C.W. and Challis, A., unpublished data). These patterns are heritable and are similar to other eucalypt species (Borralho, Cotterill, & Kanowski, 1992; Mazanec, Grayling, Spencer, Doran, & Neumann, 2017; Mora, Gleadow, Perret, & Scapim, 2009). For instance, narrow‐sense heritability for biomass in *E. loxophleba* was 0.19 ± 0.05 (Mazanec et al., 2017); height and diameter for *E. cladocalyx* were 0.28 ± 0.08 and 0.14 ± 0.10, respectively (Mora et al., 2009); and height and circumference at breast height in *E. urophylla* × *E. grandis* hybrids were 0.21 ± 0.06 and 0.14 ± 0.05, respectively (Tan, Grattapaglia, Wu, & Ingvarsson, 2018), suggesting that growth is under selection in eucalypts. Further, our findings that the difference between *Q*_ST_ and *F*_ST_ for *height* was greater than the difference between neutral *Q*_ST_ and *F*_ST_ indicates that variation in height among populations is under divergent selection and these differences are not attributable to demographic processes (e.g., genetic drift). Given similar levels of narrow‐sense heritability for both growth traits and *Q*_ST_ differentiation being significant for *height* but not for *diameter*, the growth variable *height* may be more important for local adaptation than the growth variable *diameter*.

The greater heritability for *blight resistance* in MtB could be due to higher pathogen loads, which may lead to greater trait expression. The level of heritability is congruent with *Mycosphaerella* leaf disease in *Eucalyptus globulus* (*ĥ^2^* = 0.13–0.35), which also exhibited spatial variation along a latitudinal cline (Hamilton et al., 2012). However, blight resistance in *C. calophylla* was lower than resistance to rust (*Puccinia psidii*) for *Eucalyptus dunnii*, which was moderately heritable (*ĥ^2^* = 0.37) in two trials (Pinto et al., 2014). Likewise, our heritability estimate for *blight resistance* was lower than the heritability of blight damage of the same pathogen in the related eastern Australia *Corymbia citriodora* (*ĥ^2^* = 0.31—Johnson et al., 2009; *ĥ^2^* = 0.34—Brawner et al., 2011). The lower heritability of blight resistance in *C. calophylla* could be due to the recent introduction of blight in the geographical range of *C. calophylla*, compared to long co‐evolutionary histories between the pathogen and its host in eastern Australia. However, despite the lack of a co‐evolutionary history between the blight pathogen and *C. calophylla*, the *Q*_ST_ estimate provides further evidence that trait variance is not attributable to chance or genetic drift, but is likely the result of selection pressure.

Uncovering differing levels of *blight resistance* among populations was not expected because the pathogen (*Quambalaria pitereka*) is not native to *C. calophylla*'s range in Western Australia. However, *blight resistance* appears to be genetically determined, somewhat heritable, and shows strong clinal variation, suggesting exaptation. Patterns of exaptation between naïve plants and introduced pathogens are a known phenomenon (reviewed in Newcombe & Dugan, 2010). *Corymbia calophylla* has also been shown previously to have resistance to the novel pathogen *Austropuccinia psidii* (myrtle rust; formerly *Puccinia*) (Zauza et al., 2010). Indeed, resistance to myrtle rust is variable throughout Australian Myrtaceae species (Tobias, Guest, Külheim, Hsieh, & Park, 2016; Zauza et al., 2010), and the methods of resistance have been linked to genes of large effect (*Ppr1*) in *E. grandis* (Junghans et al., 2003; Mamani et al., 2010) and SNPs linked to 13 genes that accounted for up to 70% of the variation (Thumma et al., 2013). In other families, patterns of pathogen resistance to *Cronartium ribicola* (thought to be native to eastern Asia) have been found in *Pinus* species from North America (Kinloch & Dupper, 2002). It has been argued that these patterns of resistance could be the result of “genetic memory” (Kinloch & Dupper, 2002). Genetic memory occurs when, in this case, pathogen‐resistant genes have been selected for by a related endemic pathogen, and the gene confers general resistance to the introduced pathogen. Genetic memory could explain *C. calophylla's* patterns of heritability and differentiation as similar pathogens occur throughout the distribution, most notably *Q. coyrecup* and *Q. cyanescens* (Paap et al., 2008). The presence of genetic variation within the species provides a mechanism for enhanced *blight resistance* in natural populations, and heritability provides evidence that the species is able to respond to selection pressures.

In sum, these results illustrate the genetic basis and adaptive patterns of growth and *blight resistance* among populations. Both trait types contain genetic variation and patterns of heritability, which allow for adaptive potential and independent selection. Indeed, precipitation and temperature variables can be powerful drivers of selection for these complex traits.

### 4.2 Trait–Environment correlations

Our analyses indicate that growth and defence traits in *C. calophylla* are more closely related to temperature of origin rather than precipitation of origin. The relationships between *T*_MAX_ and the three traits (*height*,* diameter* and *blight resistance*) contained more explanatory power than *P*_DM_. This finding is consistent with the significant relationship identified for leaf hydraulic vulnerability to drought (P50_leaf_) and mean annual temperature in *C. calophylla*, where mean annual precipitation was not a significant predictor (Blackman, Aspinwall, Tissue, & Rymer, 2017). Similarly, photosynthetic capacity and leaf respiration exhibited greater differences among populations from contrasting temperature‐origins compared to precipitation‐origins (Aspinwall et al., 2017). These findings also agree with a meta‐analysis by Moles et al. (2014), which found that temperature was the major driver of trait distribution in most functional traits.

The correlation between climate and traits, coupled with patterns of trait heritability, provides confidence in adaptive predictions across the landscape. While calculating models with the best possible predictive power explains a high proportion of variation, these models can be difficult to interpret due to overfitting of the data, leading to noise in future predictions (Aho, Derryberry, & Peterson, 2014). Accepting these caveats, we were able to explain the majority of the variation for *height* and *blight resistance*. Two‐factor models, with only temperature and rainfall, could also explain a majority of the variation in these traits. These models clearly show that the southern populations have faster growth strategies and higher resistance to blight, indicating that *C. calophylla* shows genetically determined patterns at the population level that confer a competitive advantage over genotypes from other climatic regions. A similar competitive advantage was identified in *Eucalyptus marginata* from Western Australia (O'Brien et al., 2017). In situ, the faster growth strategy may result in an increased ability to compete for limited resources (e.g., soil nutrients and light) during early successional stages, as fast growth strategies are known to provide a competitive advantage (Coomes & Allen, 2007; Falster & Westoby, 2003). Hence, *blight resistance* could yield a competitive advantage by maintaining maximum leaf area for the capture of light and increased production of sugars.

We predicted that *blight resistance* to the novel pathogen would be equal across all populations, but this hypothesis was rejected. The uneven distribution of *blight resistance* could be explained by increased pathogen loads in wetter climates, as *Quambalaria sp*. are more prevalent in wet conditions (above 800 mm of rainfall per year; Pegg, Carnegie, Wingfield, & Drenth, 2010). To explain the standing genetic variance in *C. calophylla* providing resistance to introduced *Q. pitereka* in cooler, wetter climates, we hypothesize that an increased prevalence of similar/related pathogens (Paap et al., 2008) could contribute to selection of traits that confer leaf blight resistance, similar to the “genetic memory” conclusion proposed by Kinloch and Dupper (2002). Regardless of the reason for blight resistance differentiation among populations, the system has the capacity to respond to selection and genotypes can be selected for future forests.

### 4.3 Management implications

In the future, the adaptive capacity of *C. calophylla* will be tested because precipitation and temperature are predicted to substantially change throughout its range (Yates et al., 2010), affecting tree performance and survival, as well as pathogen spread and load (Harvell et al., 2002). Non‐native pathogens have decimated tree populations across the globe, while pathogens continue to be introduced into different locations (Sniezko, 2006). As a key constituent of southwest Australian forests, it is imperative to proactively manage *C. calophylla* to maintain forest productivity and ecosystem services. Our research reveals differential growth strategies and blight responses in populations that can be explained by adaptation to climate. These patterns of adaptation might be exploited to increase *blight resistance* in some populations and prepare other populations for variable environmental conditions by implementing assisted gene migration or climate‐adjusted provenancing strategies (Aitken & Whitlock, 2013; Prober et al., 2015). In addition, if a breeding programme were needed in the future, *r*_p_ and *r*_g_ between traits suggests that selection of individual trees that are “correlation breakers,” exhibiting slower growth strategies and high *blight resistance*, can be created for long‐term management strategies.

## 5 CONCLUSIONS

As disturbance, pathogens and climate change reshape natural ecosystems, the ability of species to evolve and adapt to new challenges is imperative for their continued persistence. We identify standing genetic variation within a long‐lived, foundation tree species that shows differential, regional responses to climate variables and a novel pathogen. The spatial distribution of growth and *blight resistance* covaried in populations along temperature gradients, providing evidence for trait evolution. Notably, this suggests that exaptive evolutionary mechanisms for resistance to novel pathogens may be present in trees across different environments. The integration of genetic variation into adaptive management strategies, such as assisted gene migration and seed sourcing, may be used to provide greater resilience for natural ecosystems to biotic and abiotic stressors.

## CONFLICT OF INTEREST

None declared.

## DATA ACCESSIBILITY

Data for this study are available at Dryad.org https://doi.org/10.5061/dryad.gc26qb7.
