# Supplementary material for: Adaptive variation for growth and resistance to a novel pathogen along climatic gradients in a foundation tree
Source: Evol Appl. 2019 Apr 15;12(6):1178–90. doi: 10.1111/eva.12796 (PMC6597866; doi:10.1111/eva.12796)
Supplement: Supplementary file 1 [file EVA-12-1178-s001.docx]

Adaptive variation for growth and resistance to a novel pathogen along climatic gradients in a foundation tree

**Supporting information:**

Methods

**Trait variation**

Initial analyses were conducted on individual traits to assess model fit, using the following equation:

Y*_ijklmn_*= *µ* + b*_i_* + p*_j_* + br*_ik_* + bc*_il_* + t*_m_* + f*_n_* + e*_ijklmn_* .

Where Y*_ijklmn_* = individual phenotypic tree measurement, *µ* is the fixed overall mean, r_i_ is the random effect of the *j*th block, p_j_ is the random effect of the *j*th Provenance, br_ik_ is the random effect of the *k*th row nested within the *i*th block, bc_il_ is the random effect of the lth column nested within the jth block, t_m_ is the random effect of the *m*th plot representing Rep.Family interaction, f_n_ is the random effect of the *n*th family and e_ijklmn_ is the random residual error. Final estimates of variance components for each trait were conducted using the same model in multivariate form. In that analysis unstructured variance–covariance matrices were estimated for all random effects to account for association between traits.

Residual plots were examined for normality using the following equation:

y*_ijklmno_* = µ + s*_i_* + p*_j_* + sp*_ij_* + sb*_ik_*+ sbr*_ikl_* + sbc*_ikm_* + st*_in_* + f*_o_* + sf*_so_* + e*_ijklmno_* .

Where Y*_ijklmno_* = individual phenotypic tree measurement, *µ* is the fixed overall mean, s*_i_* is the fixed effect of the *i*th trial site, p*_j_* is the random effect of the *j*th provenance, sp*_ij_* is the random interaction of the *i*th site and the *j*th provenance, sb*_ik_* is the random effect of the *k*th block on the *i*th site effect, sbr*_ikl_* is the random effect of the *l*th row within the *k*th block within the *i*th site, sbc*_ikm_* is the random effect of the *m*th column within the *k*th block within the *i*th site, st*_in_* is the random effect of the *n*th plot within the *i*th site, f*_o_* is random effect of the oth family, sf*_so_* is the random interaction of the *i*th site and the *o*th family interaction and e*_ijklmo_* is the random residual error.

In order to determine if there is a genotype × environment interaction, type-B cross-site correlations (Burdon, 1977) were estimated for family effects according to the formula defined in White et al., (2007)

r_b_ = σ^2^_f_/ (σ^2^_f_ + σ^2^_sf_).

Where σ^2^_f_ is the family component of variance and σ^2^_sf_ is the family within site component of variance.

**Genotyping**

DNA extractions were performed on 20 mg of freeze dried leaf material using a modified CTAB protocol (Doyle & Doyle 1990) with 1% sodium sulfite

(Byrne *et al.* 2001) and 1% w/v polyvinylpyrrolidone (MW 40,000) added to the extraction buffer. Quality and quantity of DNA extracts were estimated using gel electrophoresis and a Qubit fluorometer (Invitrogen, Carlsbad, CA). DNA samples (400 ng) were sequenced using DArTseq™ protocols (Diversity Arrays Technology Pty Ltd, Canberra Australia) that represent a combination of a double digest complexity reduction method and next generation sequencing platforms

(Kilian *et al.* 2012). A detailed description of the DArTseq™ methodology can be found in Kilian *et al.*

(2012) and Grewe et al. (2015). Briefly, reduction of the genome was performed using a combination of PstI and HpaII enzymes in digestion/ligation reactions principally as per Kilian et al. (2012) with adapters that include individual varying length barcodes, flowcell attachment sequence, and sequencing primer, similar to Elshire et al. (2011). Raw fastq files were demultiplexed and aligned using Diversity Array Technology’s proprietary bioinformatics pipeline. Poor quality sequences filtered out of the Fastq files with a Phred pass score of 30 (probability of incorrect base is 1 in 1000). Minimum read depth for each individual was set to 6 and average read depth was 82.8 across all SNPs, ensuring call quality for all SNPs. Read lengths consisted of 75 bp and only nucleotide substitutions were considered a SNP for the SNP calling algorithm (proprietary DArTsoft14).

Further SNP filtering was performed in R (R Core Development Team 2015) using custom script. SNPs were kept if they were called in at least 175 individuals (35% missing data), however most of the SNPs (8705 of 9560 or 91%) met a 15% missing data threshold. A threshold of 35% missing data was chosen because on average there would still be data from 6-7 individuals within a population to calculate population-level allele frequency sufficient for downstream EAA analysis. Minor allele frequency was set to a minimum of 10 total calls (i.e. and allele frequency of 0.027 (2.7%)) occurring in at least 2 populations. Only one random SNP per 75 bp read was retained. Linkage disequilibrium was calculated between all SNPs and only one SNP was retained if the LD was greater than 0.5 between two SNPs. The genotype data frame was created to standards described in the hierfstat package in R (Goudet 2005).

References

Burdon, R.D. (1977) Genetic correlation as a concept for studying genotype – environment interaction in forest tree breeding. *Silvae Genetica*, 26, 168-175.

Byrne M, Macdonald B, Francki M. (2001) Incorporation of sodium sulfite into extraction protocol minimizes degradation of Acacia DNA. *BioTechniques*, 30, 742–4– 748.

Doyle J, Doyle J (1990) Isolation of plant DNA from fresh tissue. *Focus*, 12, 13–15.

Elshire, R. J., Glaubitz, J. C., Sun, Q., Poland, J. A., Kawamoto, K., Buckler, E. S., & Mitchell, S. E. (2011). A robust, simple genotyping-by-sequencing (GBS) approach for high diversity species. *PloS one*, *6*, e19379.

Goudet, J. (2005). Hierfstat, a package for R to compute and test hierarchical F‐statistics. *Molecular Ecology Notes*, *5*, 184-186.

Goudet, J. & Büchi, L. (2006) The effects of dominance, regular inbreeding and sampling design on Q_ST_, an estimator of population differentiation for quantitative traits. Genetics, 172, 1337–1347.

Grewe PM, Feutry P, Hill PL *et al.* (2015) Evidence of discrete yellowfin tuna (*Thunnus albacares*) populations demands rethink of management for this globally important resource. *Scientific Reports*, 5, 16916.

Kilian A, Wenzl P, Huttner E *et al.* (2012) Diversity arrays technology: a generic genome profiling technology on open platforms. Data production and analysis in population genomics: methods and protocols. Totowa, NJ: Humana Press, 67–89.

R Core Development Team (2015) R: A language and environment for statistical computing.

White, T.L., Adams, W.T., & Neale, D.B. (2007) Forest genetics. Trowbridge: CAB International. Cromwell Press Group.

Table S1. Current and historical experimental planting site attributes, including location, soil, and climate information. Soil attributes are shown as means of six samples from Margaret River and three samples from Mount Barker with standard error given.

|  |  | Attribute | units | Margaret River | Mount Barker |
| --- | --- | --- | --- | --- | --- |
| Geography |  | Latitude |  | -33.913 | -34.708 |
|  |  | Longitude |  | 115.191 | 118.028 |
| Climate | Historic | MAP | mm | 1039 | 670 |
|  | 1970-2000 | PreDM | mm | 11 | 25 |
|  |  | MaxT | ˚C | 26.3 | 25.9 |
|  |  | 1/ai |  | 1.09 | 1.74 |
|  | Current | MAP | mm | 1078.8 | 685.3 |
|  | 2013-16 | MaxT | ˚C | 28.3 | 29.1 |
| Soil Attributes |  | Nitrate | mg/Kg | 27.00 ±10.79 | 38.67 ±18.12 |
|  |  | P | mg/Kg | 15.57 ±2.37 | 33.33 ±6.06 |
|  |  | K | mg/Kg | 25.71±3.7 | 87.33±16.5 |
|  |  | S | mg/Kg | 12.07±1.01 | 11.83±3.23 |
|  |  | Organic C | % | 2.00 ±0.13 | 4.18 ±0.48 |
|  |  | DTPA Iron | mg/Kg | 55.03 ±10.41 | 408.46 ±35.65 |

MAP = mean annual precipitation; MaxT = Maximum Temperature of the Warmest Month; PreDM = Precipitation of the Driest Month; 1/ai = 1/ Aridity Index; P = Phosphorus; K = Potassium; S = Sulphur; Organic C = Organic Carbon; DTPA Iron = diethylenetriaminepentaacetic iron

| **Trial** | **Prov** | **Height** | **Height SE** | **Diameter** | **Diameter SE** | **Blight** | **Blight SE** |
| --- | --- | --- | --- | --- | --- | --- | --- |
| MR | LTO | 112.6 | 3.9 | 3.16 | 0.11 | 2.6 | 0.1 |
| MR | HRI | 100.8 | 4.0 | 3.08 | 0.11 | 3.0 | 0.1 |
| MR | MOG | 122.6 | 3.9 | 3.38 | 0.11 | 2.9 | 0.1 |
| MR | CRI | 123.6 | 4.2 | 3.39 | 0.12 | 3.3 | 0.1 |
| MR | EPO | 113.9 | 5.9 | 2.86 | 0.15 | 2.9 | 0.1 |
| MR | CHI | 118.5 | 3.9 | 3.34 | 0.11 | 2.8 | 0.1 |
| MR | MTB | 142.4 | 3.9 | 3.65 | 0.11 | 3.8 | 0.1 |
| MR | PEE | 115.7 | 4.0 | 3.46 | 0.12 | 3.1 | 0.1 |
| MR | WHI | 119.2 | 3.9 | 3.31 | 0.11 | 3.2 | 0.1 |
| MR | PIN | 119.9 | 3.9 | 3.45 | 0.11 | 2.9 | 0.1 |
| MR | SER | 121.7 | 3.9 | 3.37 | 0.11 | 3.1 | 0.1 |
| MR | LUP | 121.7 | 3.9 | 3.38 | 0.11 | 2.9 | 0.1 |
| MR | LEN | 133.3 | 3.9 | 3.51 | 0.11 | 3.5 | 0.1 |
| MR | GOD | 118.3 | 3.9 | 3.37 | 0.11 | 3.1 | 0.1 |
| MR | BRA | 149.8 | 4.0 | 3.88 | 0.11 | 4.0 | 0.1 |
| MR | BOO | 155.1 | 3.9 | 3.81 | 0.11 | 3.8 | 0.1 |
| MR | KIN | 136.8 | 3.9 | 3.47 | 0.11 | 3.3 | 0.1 |
| MR | CAR | 152.1 | 3.9 | 3.66 | 0.11 | 3.8 | 0.1 |
| MtB | LTO | 214.1 | 3.9 | 6.45 | 0.11 | 2.0 | 0.1 |
| MtB | HRI | 205.4 | 3.9 | 6.40 | 0.11 | 2.3 | 0.1 |
| MtB | MOG | 233.3 | 3.9 | 6.65 | 0.11 | 2.1 | 0.1 |
| MtB | CRI | 224.8 | 4.0 | 6.83 | 0.11 | 2.6 | 0.1 |
| MtB | EPO | 209.5 | 4.9 | 5.63 | 0.14 | 2.2 | 0.1 |
| MtB | CHI | 214.8 | 3.9 | 6.42 | 0.11 | 2.0 | 0.1 |
| MtB | MTB | 257.1 | 3.9 | 7.23 | 0.11 | 3.1 | 0.1 |
| MtB | PEE | 211.8 | 4.1 | 6.82 | 0.12 | 2.4 | 0.1 |
| MtB | WHI | 220.7 | 3.9 | 6.53 | 0.11 | 2.4 | 0.1 |
| MtB | PIN | 222.6 | 3.9 | 6.76 | 0.11 | 2.3 | 0.1 |
| MtB | SER | 225.2 | 3.9 | 6.54 | 0.11 | 2.5 | 0.1 |
| MtB | LUP | 235.8 | 3.9 | 6.73 | 0.11 | 2.2 | 0.1 |
| MtB | LEN | 243.3 | 3.9 | 6.94 | 0.11 | 2.8 | 0.1 |
| MtB | GOD | 224.9 | 4.0 | 6.71 | 0.11 | 2.4 | 0.1 |
| MtB | BRA | 261.0 | 4.0 | 7.60 | 0.11 | 3.3 | 0.1 |
| MtB | BOO | 280.3 | 3.9 | 7.51 | 0.11 | 3.2 | 0.1 |
| MtB | KIN | 240.5 | 3.9 | 6.79 | 0.11 | 2.6 | 0.1 |
| MtB | CAR | 262.2 | 3.9 | 7.10 | 0.11 | 3.1 | 0.1 |

Table S2. Best linear unbiased predictions (BLUPs) for the three traits from measurements taken at 23 – 25 months old within all provenances. Standard errors (SE) are given. MR = Margaret River; MtB = Mount Barker.

Table S3. Post-hoc Tukey tests (0.05) between provenances within each experimental site on measurements taken at 23 – 25 months old (MR = Margaret River; MtB = Mount Barker). Tests were done in the whole dataset (all) and within each blight resistance categorical value. Similar ranks between provenances (Prov) occur in scores between 5 – 2 and in the ‘all’ category. See figure S1 for graphical representation. There are few (PEE) differences within the highest blight load category, indicating that when blight load reaches maximum potential the height is affected. A score of 1 indicates the most severe blight incidence and 5 indicates the absence of blight. N = total number of samples. 0 = 0% affected. 1 = 1-25% affected. 2 = 26-50% affected. 3 = 51-75% affected. 4 = 76-100% affected. * lower total number of families within the EPO provenance resulting in higher standard error and thus no differences within individual *blight resistance* categories.

|  |  | **MR** | | | | | | **MtB** | | | | | |
| --- | --- | --- | --- | --- | --- | --- | --- | --- | --- | --- | --- | --- | --- |
| **Prov** | **T_MAX_** | **all** | **5** | **4** | **3** | **2** | **1** | **all** | **5** | **4** | **3** | **2** | **1** |
| BOO | 25.6 | g | a | de | d | cd | ac | i | ab | a | b | d | ab |
| CAR | 25.9 | g | ab | d | d | c | ac | hi | ab | ac | ab | cd | ab |
| BRA | 26.1 | fg | ae | de | de | ce | ac | gi | a | ab | ab | ad | ab |
| MTB | 26.7 | ef | abd | dg | cd | cd | ac | fgh | ab | ab | b | ad | ab |
| KIN | 27.7 | e | bce | efg | cd | bcd | bcd | begh | ab | ab | ab | ad | ab |
| LEN | 30.7 | ce | cd | cg | cd | cd | c | egh | ab | ab | ab | ad | b |
| CRI | 26.2 | cd | abc | bcf | ac | ac | ac | ae | ab | bc | ab | ad | ab |
| WHI | 30.0 | bd | bc | bc | ab | ac | ac | ae | ab | ab | ab | ad | ab |
| GOD | 30.2 | bd | c | bc | ac | ad | ac | ae | ab | ab | ab | abc | ab |
| PIN | 30.3 | bd | bce | ab | ac | ac | bcd | abc | ab | b | ab | ad | ab |
| PEE | 30.4 | bd | cd | ac | ac | ac | ab | ac | ab | bc | ab | ad | a |
| SER | 30.5 | bd | c | bc | bce | ac | cd | ae | ab | bc | ab | ad | ab |
| LUP | 31.6 | bd | c | ab | bc | ac | bcd | ceg | ab | ab | ab | ad | b |
| CHI | 32.2 | bd | bc | ab | ac | ade | bcd | ad | b | ab | ab | ab | ab |
| MOG | 33.3 | bd | c | bc | bc | ac | bcd | cdef | ab | ab | ab | bd | b |
| LTO | 31.2 | b | c | ab | ab | ab | ad | ac | NA | ab | ab | ad | ab |
| EPO* | 34.1 | abc | abc | acde | acd | ac | ac | abc | ab | ab | ab | ad | ab |
| HRI | 31.7 | a | c | a | a | a | a | a | ab | bc | a | a | ab |
| N |  | 3651 | 235 | 1482 | 944 | 586 | 404 | 3562 | 80 | 631 | 950 | 1158 | 743 |

Table S4. Pairwise F_ST_ estimates for each of the 18 populations. F_ST_ estimates were used in the Q_ST_ – F_ST_ comparison.

| **PROV** | BOO | BRA | CAR | CRI | LEN | HIL | CHID | HRI | KER | KIN | EPO | MOG | PLA | PEE | LTO | SER | LUP | PIN |
| --- | --- | --- | --- | --- | --- | --- | --- | --- | --- | --- | --- | --- | --- | --- | --- | --- | --- | --- |
| BOO |  |  |  |  |  |  |  |  |  |  |  |  |  |  |  |  |  |  |
| BRA | 0.035 |  |  |  |  |  |  |  |  |  |  |  |  |  |  |  |  |  |
| CAR | 0.031 | 0.035 |  |  |  |  |  |  |  |  |  |  |  |  |  |  |  |  |
| CRI | 0.043 | 0.049 | 0.048 |  |  |  |  |  |  |  |  |  |  |  |  |  |  |  |
| LEN | 0.060 | 0.045 | 0.054 | 0.067 |  |  |  |  |  |  |  |  |  |  |  |  |  |  |
| HIL | 0.064 | 0.053 | 0.057 | 0.070 | 0.039 |  |  |  |  |  |  |  |  |  |  |  |  |  |
| CHID | 0.081 | 0.068 | 0.076 | 0.084 | 0.054 | 0.051 |  |  |  |  |  |  |  |  |  |  |  |  |
| HRI | 0.102 | 0.089 | 0.097 | 0.103 | 0.077 | 0.071 | 0.048 |  |  |  |  |  |  |  |  |  |  |  |
| KER | 0.064 | 0.049 | 0.057 | 0.069 | 0.034 | 0.037 | 0.033 | 0.055 |  |  |  |  |  |  |  |  |  |  |
| KIN | 0.026 | 0.023 | 0.025 | 0.040 | 0.027 | 0.031 | 0.048 | 0.074 | 0.032 |  |  |  |  |  |  |  |  |  |
| EPO | 0.134 | 0.122 | 0.132 | 0.136 | 0.110 | 0.106 | 0.081 | 0.044 | 0.087 | 0.106 |  |  |  |  |  |  |  |  |
| MOG | 0.092 | 0.078 | 0.089 | 0.095 | 0.066 | 0.064 | 0.037 | 0.039 | 0.044 | 0.061 | 0.074 |  |  |  |  |  |  |  |
| PLA | 0.030 | 0.034 | 0.032 | 0.039 | 0.047 | 0.052 | 0.065 | 0.089 | 0.048 | 0.021 | 0.119 | 0.079 |  |  |  |  |  |  |
| PEE | 0.065 | 0.048 | 0.057 | 0.069 | 0.034 | 0.037 | 0.034 | 0.052 | 0.023 | 0.031 | 0.087 | 0.043 | 0.048 |  |  |  |  |  |
| LTO | 0.055 | 0.042 | 0.051 | 0.063 | 0.032 | 0.033 | 0.035 | 0.060 | 0.024 | 0.023 | 0.090 | 0.047 | 0.042 | 0.023 |  |  |  |  |
| SER | 0.070 | 0.054 | 0.064 | 0.073 | 0.043 | 0.041 | 0.029 | 0.051 | 0.020 | 0.039 | 0.086 | 0.042 | 0.055 | 0.025 | 0.026 |  |  |  |
| LUP | 0.063 | 0.049 | 0.059 | 0.070 | 0.038 | 0.038 | 0.031 | 0.051 | 0.021 | 0.034 | 0.084 | 0.037 | 0.049 | 0.024 | 0.025 | 0.022 |  |  |
| PIN | 0.048 | 0.038 | 0.045 | 0.058 | 0.028 | 0.031 | 0.044 | 0.069 | 0.026 | 0.019 | 0.100 | 0.057 | 0.039 | 0.025 | 0.022 | 0.032 | 0.031 |  |

Table S5. All GLMs between the growth or disease measurement and independent climate variables with their resulting r^2^ value and *P*-value.

| variable | climate | year | Exp site | intercept | slope | r^2^ | | p |
| --- | --- | --- | --- | --- | --- | --- | --- | --- |
| Height | MaxT | 2015 | MR | 141.02 | -2.1 | 0.618 | | 0.0001 |
|  |  |  | MtB | 226.9 | -2.35 | 0.437 | | 0.003 |
|  |  | 2016 | MR | 266.36 | -4.738 | 0.633 | | 0.00008 |
|  |  |  | MtB | 383.99 | -5.469 | 0.549 | | 0.0004 |
|  | PreDM | 2015 | MR | 66.64 | 0.900 | 0.561 | | 0.00035 |
|  |  |  | MtB | 141.35 | 1.180 | 0.542 | | 0.00049 |
|  |  | 2016 | MR | 98.61 | 2.020 | 0.573 | | 0.00028 |
|  |  |  | MtB | 188.24 | 2.490 | 0.565 | | 0.00032 |
|  | AI | 2015 | MR | 86.36 | -4.213 | 0.236 | | 0.04 |
|  |  |  | MtB | 167.69 | -5.801 | 0.252 | | 0.03 |
|  |  | 2016 | MR | 146.94 | -11.640 | 0.362 | | 0.008 |
|  |  |  | MtB | 246.23 | -13.490 | 0.316 | | 0.015 |
| Diameter | MaxT | 2015 | MR | 2 | -0.03 | 0.744 | | 0.000004 |
|  |  |  | MtB | 3.17 | -0.012 | 0.062 | | 0.321 |
|  |  | 2016 | MR | 4.29 | -0.06 | 0.585 | | 0.0002 |
|  |  |  | MtB | 6.31 | -0.063 | 0.412 | | 0.004 |
|  | PreDM | 2015 | MR | 1.08 | 0.010 | 0.52 | | 0.0008 |
|  |  |  | MtB | 2.76 | 0.004 | 0.05 | | 0.39 |
|  |  | 2016 | MR | 2.18 | 0.020 | 0.48 | 0.001 | |
|  |  |  | MtB | 4.14 | 0.020 | 0.28 | | 0.02 |
|  | AI | 2015 | MR | 1.34 | -0.068 | 0.466 | | 0.002 |
|  |  |  | MtB | 2.89 | -0.037 | 0.059 | | 0.3 |
|  |  | 2016 | MR | 2.86 | -0.193 | 0.572 | | 0.0003 |
|  |  |  | MtB | 4.72 | -0.145 | 0.209 | | 0.05 |
| Blight Res | MaxT | 2015 | MR | 0.03 | 0.020 | 0.22 | | 0.05 |
|  |  |  | MtB | -1.96 | 0.080 | 0.73 | | 0.000006 |
|  |  | 2016 | MR | -1.73 | 0.120 | 0.67 | | 0.00004 |
|  |  |  | MtB | -1.67 | 0.140 | 0.71 | | 0.00001 |
|  | PreDM | 2015 | MR | 0.72 | -0.005 | 0.072 | | 0.28 |
|  |  |  | MtB | 0.99 | -0.030 | 0.5 | | 0.001 |
|  |  | 2016 | MR | 2.46 | -0.045 | 0.449 | | 0.0023 |
|  |  |  | MtB | 3.18 | -0.049 | 0.435 | | 0.0029 |
|  | AI | 2015 | MR | 0.5 | 0.084 | 0.331 | | 0.01 |
|  |  |  | MtB | 0.19 | 0.206 | 0.405 | | 0.005 |
|  |  | 2016 | MR | 1.25 | 0.336 | 0.485 | | 0.001 |
|  |  |  | MtB | 1.9 | 0.341 | 0.393 | | 0.005 |

Table S6. The top results from generalised linear models with standardised data from both experimental sites. Beta coefficients for each environmental variable, the log Likelihood, AIC, and variation explained (VE). Dia = basal diameter; Blight = blight resistance; ns = not significant.

|  | 1/ai | Alt | bio1 | bio4 | bio5 | bio6 | bio8 | bio9 | bio12 | bio13 | bio14 | bio15 | bio18 | bio19 | PET | AICc | VE |
| --- | --- | --- | --- | --- | --- | --- | --- | --- | --- | --- | --- | --- | --- | --- | --- | --- | --- |
| Height | 0.35 | ns | ns | ns | ns | ns | ns | ns | 0.004 | ns | 0.08 | 0.02 | -0.02 | -0.01 | ns | -36.36 | 86.0% |
| Dia | ns | ns | ns | ns | ns | ns | ns | ns | 0.003 | ns | 0.06 | ns | -0.03 | -0.004 | ns | -29.75 | 72.7% |
| Blight | ns | ns | ns | 0.0003 | -0.02 | ns | ns | 0.02 | -0.004 | NA | -0.04 | ns | 0.02 | 0.01 | ns | -49.83 | 91.6% |

**1/ai** = 1/Aridity Index; **Alt** = Altitude; **bio1** = Annual Mean Temperature; **bio4** = Temperature Seasonality (standard deviation *100); **bio5** = Max Temperature of Warmest Month; **bio6** = Min Temperature of Coldest Month; **bio8** = Mean Temperature of Wettest Quarter; **bio9** = Mean Temperature of Driest Quarter; **bio12** = Annual Precipitation; **bio13** = Precipitation of Wettest Month; **bio14** = Precipitation of Driest Month; **bio15** = Precipitation Seasonality (Coefficient of Variation); **bio18** = Precipitation of Warmest Quarter; **bio19** = Precipitation of Coldest Quarter; **PET** = potential evapotranspiration

Figure S1. Effect of *blight resistance* on *height* across maximum temperature of the warmest month. *Blight resistance* in category 5 has the lowest number of total individuals, particularly in Mount Barker (Table S1), shown by the large standard error bars. The blight resistance scores of 2-5 show slopes that are not statistically significant from one another nor from the ‘all individuals’ figure. However, a blight resistance score of 1 shows slopes for both Trial that are statistically different then all other slopes. This describes a system in which the blight pathogen studied here does not affect the height rank until it is at its most sever (blight-1). Standard errors are given.

Figure S2. Height of provenances averaged over both trial sites (a) height, (b) diameter, and (c) blight resistance. Error bars indicate 95% confidence interval.

Figure S3. Height (a), basal diameter (b), and blight resistance (c) of provenances split by trial site (colours). Error bars indicate 95% confidence interval.
